# Determination of risk factors for predicting the onset of symptoms in asymptomatic COVID-19 infected patients

**DOI:** 10.7150/ijms.47576

**Published:** 2020-08-06

**Authors:** Pei-Yao Tao, Ling Leng, Kun Liu, Ri-Hua Zhou, Yue-Chun Hu, Shang-Jie Wu, Yu-Dong Xiao, Jun Liu

**Affiliations:** 1 Department of Radiology, The Second Xiangya Hospital, Central South University, Changsha, China, 410011.; 2 Department of Cell Biology, School of Basic Medical Science, Tianjin Medical University, Tianjing, China, 300070.; 3 Department of Infection and rehabilitation, Yiyang The Fourth People's Hospital, Yiyang, China, 413000.; 4 Medical Department, Chenzhou The Second People's Hospital, Chenzhou, China, 423000.; 5 Department of Radiology, Loudi Central Hospital, Nanhua University, Loudi, China, 417000.; 6 Department of Pulmonary and Critical Care Medicine, The Second Xiangya Hospital, Central South University, Changsha, China, 410011.

**Keywords:** COVID-19, asymptomatic infected patients, symptoms onset, risk factors, predictive value

## Abstract

**Background:** The number of asymptomatic infected patients with coronavirus disease 2019 (COVID-2019) is rampaging around the world but limited information aimed on risk factors of asymptomatic infections. The purpose of this study is to investigate the risk factors of symptoms onset and clinical features in asymptomatic COVID-19 infected patients.

**Methods:** A retrospective study was performed in 70 asymptomatic COVID-2019 infected patients confirmed by nucleic acid tests in Hunan province, China between 28 January 2020 and 18 February, 2020. The epidemiological, clinical features and laboratory data were reviewed and analyzed. Presence or absence at the onset of symptoms was taken as the outcome. A Cox regression model was performed to evaluate the potential predictors of the onset of symptoms.

**Results:** The study included 36 males and 34 females with a mean age of 33.24±20.40 years (range, 0.5-84 years). There were 22 asymptomatic carriers developed symptoms during hospitalization isolated observation, and diagnosed as confirmed cases, while 48 cases remained asymptomatic throughout the course of disease. Of 70 asymptomatic patients, 14 (14/70, 20%) had underlying diseases, 3 (3/70, 4.3%) had drinking history, and 11 (11/70, 15.7%) had smoking history. 22 patients developed symptoms onset of fever (4/22, 18.2%), cough (13/22, 59.1%), chest discomfort (2/22, 9.1%), fatigue (1/22, 4.5%), pharyngalgia (1/22, 4.5%) during hospitalization; only one (1/22, 4.5%) patient developed signs of both cough and pharyngalgia. Abnormalities on chest CT were detected among 35 of the 69 patients (50.7%) after admission, except for one pregnant woman had not been examined. 4 (4/70, 5.7%) and 8 (8/70, 11.4%) cases showed leucopenia and lymphopenia. With the effective antiviral treatment, all the 70 asymptomatic infections had been discharged, none cases developed severe pneumonia, admission to intensive care unit, or died. The mean time from nucleic acid positive to negative was 13.2±6.84 days. Cox regression analysis showed that smoking history (P=0.028, hazard ratio=4.49, 95% CI 1.18-17.08) and existence of pulmonary disease (P=0.038, hazard ratio=7.09, 95% CI 1.12-44.90) were risk factors of the onset of symptoms in asymptomatic carries.

**Conclusion:** The initially asymptomatic patients can develop mild symptoms and have a good prognosis. History of smoking and pulmonary disease was prone to illness onset in asymptomatic patients, and it is necessary to be highly vigilant to those patients.

## Introduction

Since December 2019, with the emergence and expansion of coronavirus disease 2019 (COVID-2019) or the severe acute respiratory syndrome corona virus 2 (SARS-CoV-2), the World Health Organization (WHO) has declared COVID-19 as the sixth public health emergency of international significance on 30 January 2020 1,2. As of 10 April 2020 a confirmed more than one million five hundred and ninety thousand people have been infected with COVID-2019 globally, among them, 353807 cases reported cured and 95495 cases reported deaths. After several months of containment, China has transitioned to a mitigation phase. However, the clinical manifestations of COVID-19 are protean and can present as an asymptomatic carrier state. Asymptomatic COVID-2019 infected patients were defined as follows 3-5: patients who tested (a) positive result of reverse transcription polymerase chain reaction (RT-PCR) testing for nasal or pharyngeal swab specimens twice every 24 hours; (b) without any conscious clinical symptoms prior to diagnosis, such as fever, cough, fatigue, sore throat, muscle pain, etc., and (c) the presence or absence of pulmonary pathological changes on the chest computed tomography (CT) examination after the diagnosis of infection. Early recognition of infections and cutting off the route of transmission are key points to control COVID-19. However, it is precisely because of asymptomatic and poor prevention awareness that such patients may not be given full attention and bears several challenging problems, including insidious symptom onset, subclinical manifestations and the undetectability during early stage of infection. To date, accumulated evidence has revealed that the asymptomatic carriers are infectious to an extent and can transmit COVID-19 via person-to-person contact 2,4-7. Zou, et al. 8 reported that the viral load detected in asymptomatic patients was comparable to that found in symptomatic patients, which suggested that transmission potential in asymptomatic patients was not low. Therefore, it is crucial to identify and isolate asymptomatic carriers in order to contain the outbreaks in advanced stages. However, limited data aimed on risk factors are available for asymptomatic infections. The purpose of this study is to present the risk factors of symptoms onset and clinical features in asymptomatic infected patients.

## Material and method

### Study population

This study was subject to approval by our Medical Ethical Committee (Approval No. 2020002), which waived the requirement for patients' informed consent referring to the Council for International Organization of Medical Sciences (CIOMS) guideline.

We retrospectively retrieved data for a total of 70 laboratory-confirmed positive asymptomatic carriers with COVID-19 between 28 January 2020 and 18 February 2020 by the nucleic acid in Hunan province, China. The diagnosis of COVID-19 infection based on the guidelines of China National Health Commission: Diagnosis and treatment of pneumonitis caused by new coronavirus (trial version 6) 8. Asymptomatic infections were found and quarantined in the following ways: (a) close contact with the confirmed COVID-2019 patients within 14 days; (b) with a history of travel to Wuhan or residence in epidemic areas; (c) cluster epidemic investigation; and (d) exposed population during infectious source tracking.

### Data collection

We gathered epidemiological and clinical features, laboratory examination and outcome of disease from individuals with 70 asymptomatic COVID-19 patients, via inpatient medical records in 10 designated hospitals in Hunan province, China. Basic information and demographic characteristics were collected as the following: name, age, gender, dates of diagnosis, dates of symptoms onset, and dates of each chest CT examination. Clinical information on each case during their hospitalization was also recorded the underlying disease history, smoking and drinking history, the first onset of symptoms during quarantined and the corresponding dates. The definition of positive chest CT imaging findings was that the patients were presence of the CT imaging features that favored viral pneumonia such as ground-glass opacity (GGO), bilateral patchy shadowing, local patchy shadowing, as well as interstitial abnormalities 8. Laboratory tests were conducted after admission, including leukocyte count (WBC), lymphocyte count (LY), neutrophil ratio (NUET), and lymphocyte ratio (LYM). The retrospective study began with the diagnosis of asymptomatic infection with COVID-2019, and absence or presence of symptoms was taken as the outcome. For patients with symptoms of onset, the follow-up time was the interval between the date of symptoms onset and the date of nucleic acid positive, while for remained asymptomatic carriers was the time interval between nucleic acid positive and negative. Fitness for discharge was based on the absence of obvious symptoms, with improved evidence on chest CT and the results of two real time RT-PCR tests taken 24 hours apart were negative for COVID-2019 antigens.

### Statistical analysis

Baseline demographics and clinical characteristics were compared between asymptomatic infected patients' illness onset or not. Results are presented as mean with standard deviation (SD) or frequency. Pearson's chi-square test or Fisher's exact test was applied to continuous variables or categorical variables. A multivariate Cox regression model was performed to evaluate the potential predictors of COVID-2019 asymptomatic infected patients' illness onset. Variables with *P*<0.1 in the univariate analysis and those closely related to symptom onset were included in the multivariate Cox regression model. All statistical analyses were conducted with SPSS version 25 (International Business Machines Corporation, New York, the Unites States), and *P*<0.05 was considered statistically significant.

## Results

### Demographic characteristics

The study population included 36 males and 34 females and individuals of all ages were involved in the asymptomatic COVID-19 infection with a mean age of 33.24±20.40 years (ranging from 0.5-84 years). 5 (5/70, 7.1%) of the 70 asymptomatic quarantined patients were associated with familial clusters, 3 (3/70, 4.3%) closely contacted with the confirmed patients, and the rest (62/70, 88.6%) were the exposed population during the tracking of infectious source. 14 cases (14/70, 20%) had coexisting disorders—6 (6/70, 8.6%) had hypertension, 5 (5/70, 7.1%) had diabetes, and 3 (3/70, 4.3%) had pulmonary disease. A total of 11 (11/70, 15.7%) cases had smoking history, and 3 (3/70, 4.3%) cases had a history of drinking.

### Symptoms

**Figure 1:**
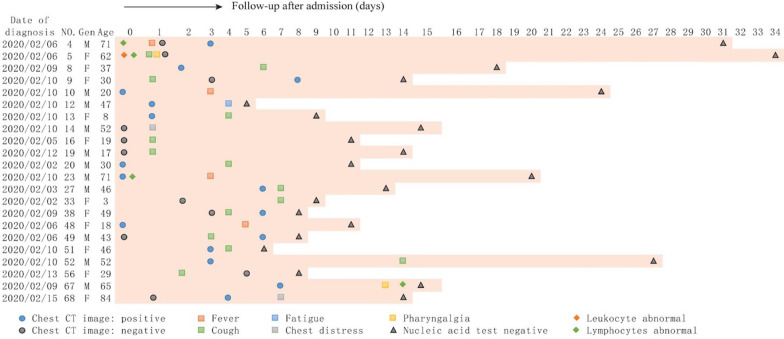
The clinical characteristic of the 22 symptomatic patients. Date of diagnosis (nucleic acid test positive) was defined as origin point, abnormal blood test and chest CT image upon admission were listed; and symptoms and nucleic acid test were recorded during hospitalization.

**Figure 2:**
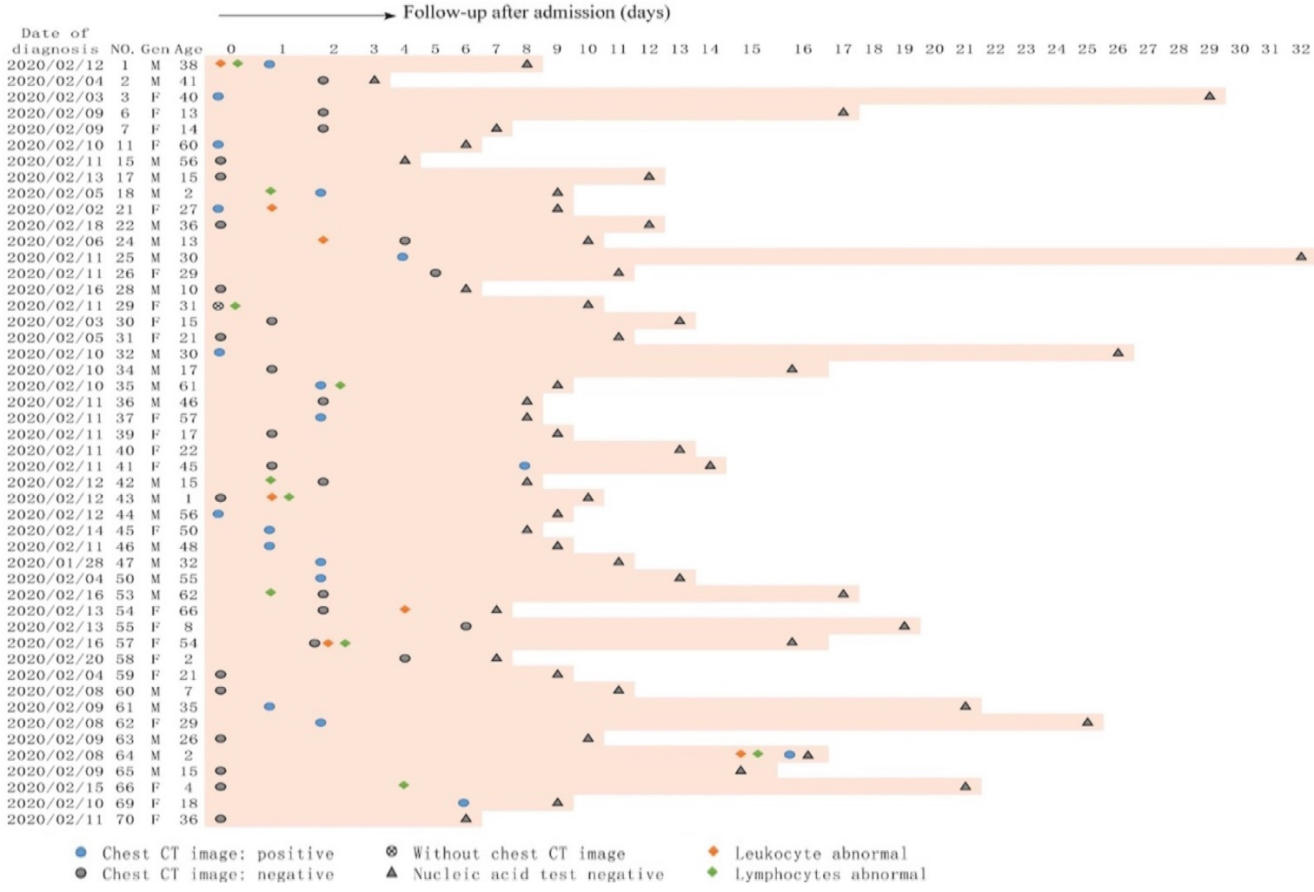
The clinical characteristic of the 48 patients who remained asymptomatic during hospitalization. Date of diagnosis (nucleic acid test positive) was defined as origin point, abnormal blood test and chest CT image upon admission were listed; and nucleic acid test were recorded during hospitalization.

Totally, 22 asymptomatic patients developed symptoms during hospitalization, whereas 48 patients remained asymptomatic throughout the course of the disease. The initial symptoms at onset of illness in 22 patients were fever (4/22, 18.2%), cough (13/22, 59.1%), chest discomfort (2/22, 9.1%), fatigue (1/22, 4.5%), pharyngalgia (1/22, 4.5%); only one (1/22, 4.5%) patient first developed cough and pharyngalgia at the same time. The mean time from nucleic acid positive to symptoms onset was 4.41±3.55 days (ranging from 1-14 days). The details are shown in **Figures 1 and 2.**

### Radiologic and laboratory findings

Abnormalities on chest CT were detected among 35 of the 69 patients (35/69, 50.7%) after admission, except for one pregnant woman who had no symptoms and not meant examined. The images of 35 patients with positive lung CT were diverse, among which GGO was the most common (28/35, 80.0%), followed by interstitial abnormalities (3/35, 8.6%), local patchy shadowing (2/35, 5.7%) and subpleural shadowing (2/35, 5.7%). In addition, 15 patients (15/35, 50.1%) had bilateral involvement. The mean time from positive tests with nucleic acid to abnormalities on chest CT was 2.91±3.35 days (ranging from 0-16 days). After admission, 4 (4/70, 5.7%) and 8 (8/70, 11.4%) cases showed leucopenia (white blood cell count <3.5×10^9^ /L) and lymphopenia (lymphocyte count <1.1×10^9^ /L). The detailed information of clinical characteristics and laboratory results are listed in **Table 1.**

### Treatment and clinical outcome

Antiviral therapy was given to all 70 cases as initiated therapy, among which 6 (6/70, 8.6%) and 2 patients (2/70, 2.9%) received interferon atomization and lopinavi alone respectively, and the other 62 patients (72/70, 88.6%) received more than 2 antiviral treatments (The details are shown in **Table 1**). After an effective treatment, none of the cases developed severe pneumonia or admission to intensive care unit, and none died. As of February 18, 2020, all the 70 asymptomatic COVID-19 infected patients had been discharged.

### Potential predictors of asymptomatic patients' illness onset

The mean follow-up time was 9.93±6.66 days, ranging from 1-32 days. The mean time from nucleic acid positive to negative was 13.2±6.84 days (range, 3-34 days). Age (χ^2^=4.51, *P*=0.036), pulmonary disease history (χ^2^=2.99, *P*=0.104), smoking history (χ^2^=5.42, *P*=0.026), drinking history (χ^2^=4.61, *P*=0.044), and positive chest CT scan (χ^2^=4.66, *P*=0.038) of asymptomatic carriers were statistically different in the univariate Cox regression model, these factors as well as interested factors, including gender, leukocyte and lymphocyte classification were embraced in the multivariate Cox regression analysis. The results showed that history of smoking (*P*=0.028, hazard ratio=4.49, 95% CI: 1.18-17.08) and pulmonary disease (*P*=0.038, hazard ratio=7.09, 95% CI: 1.12-44.90) were predictors of the onset of symptoms in asymptomatic carries with COVID-19 (**Table 2**).

## Discussion

Currently, the number of asymptomatic infected patients with COVID-19 is rampaging around the world and previous evidence has pointed out human to human transmission, which plays a minor role and intensities the difficulty of prevention and management in the epidemic overall. Owing to lack of distinct clinical symptoms for asymptomatic carriers in the initial stage of infection, the disease information may be not provided with full attention. The current study presented clinical characteristics and evaluated the potential factors for symptoms onset in asymptomatic COVID-19 infected patients during quarantined. The results showed that the clinical manifestations and laboratory examinations of asymptomatic patients were nonspecific, and had a good prognosis. History of smoking and pulmonary disease were risk factors of symptoms onset in asymptomatic carriers. Therefore, in order to better predict and control the development of disease, it is necessary to identify and isolate asymptomatic infections at an early stage, and to consider the impact of individuals' heterogeneity on the course of the disease.

There 11 and 3 asymptomatic carriers presented history of smoking and pulmonary disease respectively in the current study. Although the risk factors of COVID-19 remain unclear, previous studies reported that a significant proportion of infected patients had underlying diseases 3,9-12. Guo L, et al designed a predictive tool using the MuLBSTA score system, and revealed age, hypertension, smoking history, namely multilocular infiltration, lymphopenia, and bacterial co-infection were 6 poor prognostic factors in patients with viral pneumonia 13. Further investigation is required to explore the applicability of the MuLBSTA score in predicting the risk of COVID-2019 infection. Another study showed the indicators of disease severity associated with poor clinical outcomes, including leukocyte and lymphocyte count, oxygenation, respiratory rate, and chest imaging findings 10. In summary, previous studies suggested that patients who were older, with underlying diseases, and history of smoking were more likely to increase disease severity and predict a poor outcome 9-12,14. However, these results need further clarification, since the reported subjects were all confirmed symptomatic COVID-2019 patients, and most of the reported individuals had died or remained hospitalized before the results publication.

The present study showed that initial signs of asymptomatic infected patients were cough, followed by fever. As we all know, the virus particles can spread through the respiratory mucosa, act on T lymphocytes and induce cytokine storm in the body, give rise to a series of immune responses, and cause changes of immune cells such as peripheral blood leukocytes and lymphocytes. In terms of laboratory tests, most confirmed COVID-2019 patients showed lymphopenia, and normal or decreased leukocyte count 5,10,13. A prospective study of 61 COVID-2019 patients, including 44 mild and 17 moderate to severe patients, found that neutrophil to lymphocyte ratio (NLR) was a reliable early marker of severe progression in COVID-19 patients 14. However, all of these patients were already showing varying degrees of symptoms at the time of diagnosis and most of them were still hospitalized, with conditions may change during follow-up, and the study has not included the ultimate survival outcomes.

Indeed, the chest CT imaging is important for screening asymptomatic COVID-19 patients and aiding quarantine and infection control strategies. However, due to differences among individual and disease severity, the onset of symptoms do not necessarily present with chest CT abnormalities, as previous study described 15. Negative of chest CT imaging finding may reflect that the disease is still in an early stage and repeated subsequent CT scan is always needed to determine changes of disease. However, current study showed that chest CT findings negative and laboratory tests were not risk factors for symptomatic attack in asymptomatic patients, which may be associated with the exposure time of patients, individual immune status, and improvement in the patient's condition after rapid treatment during hospitalization. Therefore, the asymptomatic infection patients should be screened and sampled at the same time, nucleic acid detection and chest CT scans should be carried out as early as possible. Moreover, growing evidences are showing that lung ultrasound may be considered a useful alternative to chest CT for diagnosis and management of COVID-19 given its no X-ray exposure, bedside application, reduced exposition between healthy and infected patients, and was repeatable for follow-up, particularly in pregnant women 16-18.

After an effective antiviral treatment, all the 70 asymptomatic infections had been discharged. The common characteristics of those asymptomatic carriers tended to be younger, with a mean age of 33.24±20.40 years, and the mean age was markedly higher in cases of developed symptoms than remained asymptomatic cases (40.86±21.85 vs 29.74±18.92). The result may be attributed to the following: (a) due to individual differences such as age and physical condition, for asymptomatic carriers with strong immunity and physical fitness, particularly who remained asymptomatic throughout the course of disease, there is a certain degree of infectivity but may be less pathogenicity, or body's immune system can kill the virus; (b) for those who are still in the incubation period without symptoms, the human body and the virus may have reached a kind of new symbiotic state. The virus can multiply in the body and spread to the outside world, and become an important source of infection. The combination of prevention and control in China has enabled the asymptomatic infections to receive timely treatment, early isolation, examination and treatment, which contain the progress of the disease effectively.

There are several limitations in the present study. First, due to the retrospective nature, only 70 asymptomatic carriers were included during the study period. However, these estimates will be refined as the number of affected asymptomatic carriers is rampaging and being collected. Second, as the patients were only from Hunan province, it would be better to include as many asymptomatic carriers as possible in other cities in China, and even other countries to get a more comprehensive understanding of asymptomatic carriers with COVID-2019. Further validation is needed in larger multi-institutional studies. Third, lung ultrasound can be used to monitor lung involvement during a specific treatment and can be easily used in every setting, as previous study described, but we lack of lung ultrasound studies. Lastly, the date of nucleic acid test positive to negative may be shorter than the 10-day hospitalization, which could result in biases of clinical observation features.

In conclusion, COVID-19 can present as asymptomatic carriage. The clinical manifestations of initially asymptomatic patients presented mild symptoms and a good prognosis. Histories of smoking and pulmonary disease were prone to illness onset in asymptomatic infected patients with COVID-19, and more attention should be paid for those individuals. Further studies are needed to elucidate the clinical characteristics, risk and prognostic factors of asymptomatic carriers.

## Figures and Tables

**Table 1:** Personal and clinical characteristics of 70 asymptomatic COVID-19 infected patients in Hunan province, China

	All asymptomatic patients no. (%), (N=70)	Remain asymptomatic no. (%), (N=48)	Symptom onset no. (%), (N=22)	*P* value
**Age mean (years)**	33.24±20.40	29.74±18.92	40.86±21.85	0.034
**Gender**				1
Male	36 (51.4)	25 (52.1)	11 (50.0)	
Female	34 (48.6)	23 (47.9)	11 (50.0)	
**Coexisting conditions**				
Hypertension	6 (8.6)	3 (6.3)	3 (13.6)	0.37
Diabetes	5 (7.1)	3 (6.3)	2 (9.1)	0.646
Pulmonary disease	3 (4.3)	1 (2.1)	2 (9.1)	0.231
Drinking history	3 (4.3)	0	3 (13.6)	0.028
Smoking history	11 (15.7)	4 (8.3)	7 (31.8)	0.029
**Initial symptoms during quarantine**				
Fever	4 (5.7)		4 (18.2)	
Cough	13 (18.6)		13 (59.1)	
Chest distress	2 (2.9)		2 (9.1)	
Fatigue	1 (1.4)		1 (4.5)	
Muscle pain	0		0	
Pharyngalgia	1 (1.4)		1 (4.5)	
Cough and pharyngalgia	1 (1.4)		1 (4.5)	
**Time interval from RT-PCT positive to symptom onset mean (days)**			4.41±3.55	
Chest CT scan abnormal	35 (50.7)	19 (40.4)	16 (72.7)	0.019
Ground-glass shadow	28 (40.0)	14 (29.2)	12 (54.5)	
Interstitial abnormalities	3 (4,.3)	2 (4.2)	1 (4.5)	
Local patchy shadowing	2 (2.9)	1 (2.1)	1 (4.5)	
Subpleural shadowing	2 (2.9)	0	2 (18.2)	
Time from positive chest CT scan to RT-PCR tests mean (days)	2.91±3.35	2.74±3.84	3.13±2.75	0.732
Time from positive to negative tests with RT-PCR mean (days, range)	13.20±6.84 (3-34)	12.46±6.23 (3-32)	14.82±7.94 (5-34)	0.18
Leucocytes count mean (× 10^9^/L)	6.09±1.79	6.20±1.96	5.84±1.32	0.435
Increased (>9.5)	4 (5.7)	4 (8.3)	0	
Normal range (3.5-9.5)	62 (88.6)	41 (85.4)	21 (95.5)	
Decreased (<3.5)	4 (5.7)	3 (6.3)	1 (4.5)	
Lymphocytes count mean (× 10^9^/L)	2.66±5.97	2.06±1.02	3.96±10.59	0.216
Increased (>3.2)	6 (8.6)	5 (10.4)	1 (4.5)	
Normal range (1.1-3.2)	56 (80.0)	38 (79.2)	18 (81.8)	
Decreased (<1.1)	8 (11.4)	5 (10.4)	3 (13.6)	
**Epidemiology**				
Familial cluster	5 (7.1)	4 (8.3)	1 (4.5)	
Close contact history	3 (4.3)	3 (6.3)	0	
Exposure history	62 (88.6)	41 (85.4)	21 (95.5)	
**Treatment**				
Interferon atomization	6 (8.6)	2 (4.2)	4 (18.2)	
Interferon atomization + Arbidol hydrochloride	5 (7.1)	4 (8.3)	1 (4.5)	
Interferon atomization + Oseltamivir	1 (1.4)	1 (2.1)	0	
Interferon atomization + Ribavirin	1 (1.4)	1 (2.1)	0	
Interferon atomization + Lopinavi	33 (47.1)	22 (45.8)	11 (50)	
Interferon atomization + Lopinavir + Arbidol hydrochloride	8 (11.4)	4 (8.3)	4 (18.2)	
Interferon atomization + Lopinavir + Ribavirin	13 (18.6)	12 (25)	1 (4.5)	
Lopinavi	2 (2.9)	1 (2.1)	1 (4.5)	
Lopinavir + Arbidol hydrochloride	1 (1.4)	1 (2.1)	0	
**Clinical outcome**				
Discharged	70 (100)	48 (100)	22 (100)	

Note: All values are expressed as the mean ± standard deviation or number (%) as appropriate according to the primary studies.

**Table 2:** The risk factors of COVID-2019 asymptomatic infected patients' illness onset

	Univariate analysis		Multivariate analysis	
	OR (95% CI)	*P*-value	HR (95% CI)	*P*-value
Age	1.02 (1.00-1.04)	0.036	1.01 (0.98-1.04)	0.372
Gender (male vs female)	1.05 (0.46-2.43)	0.904	0.25 (0.06-1.04)	0.056
**Coexisting conditions**				
Hypertension	0.49 (0.14-1.67)	0.254		
Diabetes mellitus	0.67 (0.16-2.89)	0.595		
Pulmonary disease	0.30 (0.07-1.28)	0.104	7.09 (1.12-44.90)	0.038
Drinking history (presence vs absence)	0.29 (0.08-0.97)	0.044	2.50 (0.60-10.35)	0.207
Smoking history (presence vs absence)	0.36 (0.14-0.88)	0.026	4.49 (1.18-17.08)	0.028
Chest CT scan positive (presence vs absence)	0.37 (0.14-0.95)	0.038	1.66 (0.50-5.51)	0.411
Leucocytes count (decreased vs without decreased)	1.20 (0.16-8.93)	0.858	0.59 (0.06-5.31)	0.637
Lymphocytes count (decreased vs without decreased)	0.81 (0.24-2.75)	0.742	1.79 (0.37-8.69)	0.473

## Abbreviations

COVID-2019: coronavirus disease 2019
SARS-CoV-2: severe acute respiratory syndrome corona virus 2
WHO: World Health Organization
RT-PCR: reverse transcription polymerase chain reaction
CT: computed tomography
CIOMS: Council for International Organization of Medical Sciences
GGO: ground-glass opacity
WBC: leukocyte count
LY: lymphocyte count
NUET: neutrophil ratio
LYM: lymphocyte ratio
SD: standard deviation
NLR: neutrophil to lymphocyte ratio
